# Vorasidenib in IDH1/2-mutant low-grade glioma: the grey zone of patient’s selection

**DOI:** 10.3389/fonc.2023.1339266

**Published:** 2024-01-11

**Authors:** Lidia Gatto, Vincenzo Di Nunno, Alicia Tosoni, Stefania Bartolini, Lucia Ranieri, Enrico Franceschi

**Affiliations:** ^1^Department of Oncology, Azienda Unità Sanitaria Locale (AUSL) Bologna, Bologna, Italy; ^2^Nervous System Medical Oncology Department, IRCCS Istituto delle Scienze Neurologiche di Bologna, Bologna, Italy

**Keywords:** vorasidenib, low grade glioma, IDH 1/2 mutation, IDH gliomas mut, IDH mutation

## Opinion

Mellinghoff et al. (1) have published the practice-changing results of the phase 3 INDIGO trial reporting that vorasidenib, a dual IDH1/2 inhibitor, significantly improves the progression free survival (PFS) and prolong the time to next intervention (TTNI) in a cohort of young adults with recurrent or progressive IDH1/2-mutant grade 2 gliomas.

Among primary brain tumors, low-grade gliomas, present multifaceted histological and biological variability, and predominantly involve young adults up to 50 years of age (2). Hotspot point mutations in IDH1/2 occur in the vast majority of adult low-grade gliomas (3) representing a potential therapeutic target since IDH mutation is an early oncogenetic event, stable overtime, whose molecular downstream pathway is well known. The current post-surgery standard of care for IDH-mutated low-grade gliomas, in patients classified as “high risk”, consists of a combined adjuvant chemo-radiotherapy regimen, associated with short- and long-term toxicity, but which guarantees a long period of clinical and radiological remission; patients classified as “low risk”, instead, do not receive adjuvant treatment and are subjected to periodic clinical and radiological observation (4, 5).

Vorasidenib is an oral inhibitor of mutant IDH1 and IDH2 glioma cells, specifically designed for brain penetrance, which showed consistent suppression of D-2-hydroxyglutarate (2-HG), the oncometabolite that drives cell proliferation (6) and favors brain-tumor related epilepsy.

The INDIGO trial involved 331 patients with recurrent/residual grade 2 astrocytoma/oligodendroglioma IDH-mutated, randomized 1:1 to receive vorasidenib 40 mg or placebo. Key eligibility criteria included a Karnofsky performance-status score of at least 80, previous surgery (with the most recent surgery occurring within 1 to 5 years) but no other anticancer treatment, measurable non-enhancing tumor, no need of immediate adjuvant chemoradiotherapy (in the judgment of the clinician). Exclusion criteria included high-risk features (such as disease with contrast enhancement on MRI, brain-stem involvement or uncontrolled disease-related symptoms). Median PFS was 27.7 months for vorasidenib versus 11.1 months for placebo, TTNI resulted not reached for vorasidenib versus 17.8 months for placebo arm. Vorasidenib had a manageable safety profile. Adverse events in the treatment group, for the most part, proved to be manageable and resolvable. Elevation of the hepatic enzyme alanine aminotransferase was the most common grade ≥3 adverse event and occurred in 9.6% of patients receiving vorasidenib (1).

The impact of vorasidenib on health-related quality of life (HRQoL), assessed by Functional Assessment of Cancer Therapy-Brain (FACTBr) questionnaire, was a secondary endpoint. Exploratory endpoints included neurocognitive outcome, assessed by validated cognitive performance instruments, and seizure frequency and severity, assessed using a patient diary. Recent additional data from the INDIGO study indicate that vorasidenib allows the preservation of HRQoL (7) and is effective across IDH-mutant gliomas with various additional mutations (8). In particular, no clinically meaningful deterioration of HRQoL has been observed in both arms (7). The issue of seizure control in patients with low-grade glioma is crucial. In fact, low-grade gliomas exhibit high rate of epileptogenicity (9, 10) because 2-HG mimics the action of glutamate on N-methyl-D-aspartate receptors (NMDA) receptors, increasing the electrical activity of neurons (11, 12); therefore, targeting IDH can impact on the personalized management of glioma-associated epilepsy, improving seizure control (13, 14). At baseline, active seizures (≥ 1 seizure in the previous 30 days) were reported in 20/168 patients (11.9%) in vorasidenib arm and in 20/163 patients (12.3%) in placebo arm. On-treatment seizure frequencies and neurocognitive function will be presented by arm (7).

The value of these results is of great impact: targeting IDH is the first successful attempt to apply “precision oncology” in low-grade gliomas, with several positive implications. First, IDH inhibition entails blocking gliomagenesis and dedifferentiation mechanisms; moreover, delaying radiotherapy implies delaying the onset of neurocognitive disorders over time, which significantly compromise young patients’ quality of life and social relationships (15, 16).

The implications of this new therapy in terms of prolonging the time to progression and improving the quality of life are noteworthy, but despite the great enthusiasm for this study, the selection of patients eligible to receive vorasidenib remains a critical point. The main questions are: who is this treatment for? Do we strictly need to apply the RTOG (4) and EORCT (5) risk criteria for deciding if patients should be immediately treated with radio and chemotherapy? Is it the real-world experience? The answers are not unique.

According to what stated by the authors and with the inclusion criteria of the study, “the patient population in the trial represents the earliest clinical phase in tumorigenesis of IDH-mutant WHO grade 2 glioma”, or rather patients classified as “low risk” and currently candidate for a “watch and wait” approach. This means that in the subgroup of low-risk patients we can identify a grey area, which we might define “intermediate risk”, represented by patients who can benefit most from vorasidenib. These are mainly patients who, after surgery, in the absence of important unfavorable prognostic factors, present a residual or recurrent non-enhancing disease, stable or slowly growing, which does not require immediate radiotherapy or chemotherapy treatment: however, this evaluation, which implies the subjective judgment of the clinician, leaves a margin of doubt and uncertainty.

Perhaps the time has come to abandon the old binary risk stratification (“low-risk” versus “high-risk”), which still contains arbitrary elements (like the age cut-off), proving impractical in real-world clinical decision-making, and to adopt a new one, also taking into account many emerging prognostic biomarkers (BRAF V600E mutation, methylome, transcriptome sequencing) (17).

Future “*ad hoc*” studies, clinical practice and incorporation of ongoing trials results (CODEL, CATNON, NRG-BN005) will refine these selection criteria.

Some further points still need to be clarified: how to treat enhancing tumors? Is it reasonable to believe that enhancing tumors are biologically different from low-grade IDH-mutated tumors and are therefore resistant to the action of IDH inhibitors?

Should we consider the possibility of integrating different treatments, for example surgical resection of the enhancing area of the tumor and pharmacological treatment with vorasidenib of the remaining lower-grade non-enhancing disease?

What will be the duration of the treatment? And what impact could this long-term therapy have on family planning and the fertility of such young patients? These questions are still under debate.

Another interesting field of research is the study of drug combinations, to evaluate the effect of combining IDH-inhibitors with chemoradiation treatments in patients with WHO grade 3 or 4 IDH-mutant gliomas.

Currently available data does not support evidence for use in “high risk” low-grade glioma or in high grade glioma; while the translational analyzes of the INDIGO trial are underway and a more mature follow-up is awaited, further clinical trials are needed to establish the role of this class of drugs in high-grade gliomas, in patients pre-treated with chemoradiotherapy or in association with other therapies.

In conclusion, the results of INDIGO trial are expected to set a new standard of care of previously untreated low grade IDH 1/2 mutant diffuse glioma not in immediate need for other intervention such as radiotherapy or chemotherapy. Finally, IDH inhibitors also find space in brain tumors as well as in the hematological field (acute myeloid leukemia with IDH1/2 mutation) and in that of advanced cholangiocarcinoma. This is the first targeted therapy for these tumors and is probably the most important advance in the treatment of low-grade gliomas in the last decade.

The interest of neuro-oncology in the development of IDH inhibitors in different disease settings (for example high-risk patients or enhancing high-grade gliomas) and/or in association with other anti-cancer agents is very high: several phase I trials are underway, aiming to evaluate the efficacy of vorasidenib in combination with tumor specific peptide vaccine (NCT05609994) and in combination with pembrolizumab (NCT05484622).

Gaining an understanding whether it is expected in the near future is very exciting: identifying biomarkers predictive of treatment response or resistance, establishing IDH inhibitors effectiveness in mitigating seizure risk, getting the follow-up data with respect to overall survival and evaluating the possibility of combination strategies with chemoradiation or immunotherapy are the next steps long awaited.

## Author contributions

LG: Conceptualization, Methodology, Writing – original draft, Writing – review & editing. VD: Validation, Writing – review & editing. AT: Validation, Writing – review & editing. SB: Validation, Writing – review & editing. LR: Validation, Writing – review & editing. EF: Supervision, Writing – review & editing.
